# Efficacy and Safety Profile of Ticagrelor Versus Clopidogrel in Percutaneous Coronary Intervention (PCI) for Acute Coronary Syndrome (ACS): A Systematic Review and Meta-Analysis

**DOI:** 10.7759/cureus.46455

**Published:** 2023-10-04

**Authors:** Shahzaib Maqbool, Muhammad Sajjad Ali, Abdur Rehman, Mohammad Ebad Ur Rehman, Javed Iqbal, Azeen Razzaq, Amer Kamal, Shivani Shivamadhu Shivamadhu, Maham Afzal, Faizan Fazal, Jawad Basit, Syed Aizaz Khalid

**Affiliations:** 1 Department of Cardiology, Rawalpindi Medical University, Rawalpindi, PAK; 2 Department of Neurosurgery, Mayo Hospital, Lahore, PAK; 3 Department of Medicine, The University of Jordan, Amman, JOR; 4 Department of Medicine, Ramaiah Medical College, Bengaluru, IND; 5 Department of Medicine, Fatima Jinnah Medical University, Lahore, PAK; 6 Department of Forensic, Federal Medical College, Islamabad, PAK

**Keywords:** cardiology research, ticagrelor, percutaneous coronary intervention (pci), acute coronary syndrome (acs), clopidogrel

## Abstract

The utilization of individualized anti-platelet therapy is of paramount significance in this era of cardiovascular advancement. This meta-analysis is also aiming to get more information relating to the effectiveness of ticagrelor versus clopidogrel among patients undergoing percutaneous coronary intervention (PCI) for acute coronary syndrome (ACS). A comprehensive literature search was done through various databases like PubMed, Google Scholar, EMBASE, Web of Science, and the Cochrane Database Library from January 15, 2023, to February 23, 2023. After careful screening, eight articles with highly significant variables were involved in the synthesis of this meta-analysis. Data analysis was done through Review Manager (RevMan, Version 5.4; The Cochrane Collaboration, Copenhagen, Denmark). In our study, ticagrelor and clopidogrel were evaluated in 10614 and 14662 patients, respectively. Ticagrelor was significantly superior to Clopidogrel in terms of all-cause mortality (RR 0.79, 95% CI 0.69-0.91, p = 0.001), risk of MI (RR 0.74, 95% CI 0.61-0.89, p = 0.001), and stroke (RR 0.64, 95% CI 0.42-0.98, p = 0.04), but a higher risk of bleeding events was observed with Ticagrelor (RR 1.36, 95% CI 1.04-1.79, p = 0.03). The two regimens were comparable in terms of stent thrombosis. Ticagrelor was found to be best in terms of reducing post-PCI myocardial infarction, stroke, stent thrombosis, and all other mortality events in comparison to Clopidogrel. However, the bleeding events were of significant concern for the utilization of ticagrelor and required further investigations.

## Introduction and background

In the modern landscape of cardiology, the utilization of dual anti-platelet therapy (DAPT) is considered a cornerstone in the long-term prevention of ischemic events and mortality risk among patients undergoing percutaneous coronary intervention (PCI) for acute coronary syndrome (ACS) [1]. The use of potent oral P2Y12 inhibitors is now widely recommended to counter inadvertent cardiovascular events. The use of ticagrelor in combination therapy with aspirin is widely recommended as a dual anti-platelet therapy for the treatment and prophylaxis of cardiovascular events [2]. However, the risk of bleeding may sometime outweigh the benefits of this dual therapy [3]. The utilization of individualized anti-platelet therapy is of paramount significance in this era of cardiovascular advancement.

The literature has depicted the effectiveness of DAPT with aspirin and P2Y12 inhibitors with significant platelet inhibition, but newer P2Y12 inhibitors (Ticagrelor and Prasugrel) are proving to be more effective with consistent platelet inhibition as compared to Clopidogrel [4]. Now, various guidelines are recommending the use of ticagrelor and prausogrel in the treatment of acute coronary syndrome with or without percutaneous coronary intervention [5]. In the same vein, the ethnic variation in side effect profiles and benefits between the western population and the east Asian population is also posing a difficulty in the selection of favorable anti-platelet therapy. The utilization of newer P2Y12 inhibitors is found to pose a greater bleeding risk among the Asian population, as depicted in various literature [6,7].

In a trial-based study named PLATO (platelet inhibition and patient outcomes), Ticagrelor showed superior results in terms of cardiovascular events, myocardial infarction, stent thrombosis, and death with a minor risk of bleeding complications as compared to Clopidogrel [8]. There are different findings from different studies, and still there are lots of discrepancies leading to the exact usefulness of newer P2Y12 inhibitors and Clopidogrel. This meta-analysis is also aiming to get more information relating to the effectiveness of ticagrelor versus clopidogrel among patients undergoing percutaneous coronary intervention for acute coronary syndrome. 

## Review

Methods

Search Strategy

This study was conducted in accordance with Preferred Reporting Items for Systematic Reviews and Meta-Analyses (PRISMA) guidelines [9]. A comprehensive literature search was done from January 15, 2023, to February 23, 2023. The literature search was conducted through five different databases, like PubMed, Google Scholar, EMBASE, Web of Science, and the Cochrane Database Library. The literature search was done through various MeSH terms of paramount significance, such as “Clopidogrel” and “Newer P2Y12 inhibitors” and “Ticagrelor” and “Percutaneous Coronary Intervention” and “Acute Coronary Syndrome." The trial studies were also included in the synthesis of this systematic review, and searches of various trials were done using the ClinicalTrials.gov website.

Study Selection

A pre-defined inclusion (studies with patients age ≥ 18 years and diagnosed with acute coronary syndrome for which they underwent percutaneous coronary intervention, both randomized controlled and observational studies) and exclusion criteria (missing data such as data related to medical history, outcome variables of interest and demographics, and not fulfilling the standard criteria of acute coronary syndrome) was established. The study selection was done by two potential authors (M.S.H and A.R). The study selection was done through the assessment of relevant titles, abstracts, and retrieved references, and those not falling under inclusion criteria were excluded. The full-text articles retrieved after the selection process were then assessed by two independent authors, and any dispute among them was solved with the help of a third author (S.M). The PICO criteria of the study are given in Table 1. 

**Table 1:** Showing the PICO criteria of the study among patients undergoing percutaneous coronary intervention for acute coronary syndrome

Variable	Characteristic
Population	Patients with acute coronary syndrome (ACS)
Intervention	Ticagrelor (180-mg loading dose, 90-mg twice daily thereafter for 12 months)
Comparator	Clopidogrel (300-to-600mg loading dose, 75 mg daily for 12 months)
Outcomes	All-cause mortality post-PCI myocardial infarction stent thrombosis stroke, bleeding events

Data Extraction

The standard variables of interest like author name, year of study, country of study, mean age of the patients, and study type were extracted in the first place, and disease-specific variables of interest like disease characteristics, type of drug given, follow-up duration, cardiovascular events, myocardial infarction, death, risk of bleeding, and finally the type of procedure patients were undergoing will be extracted.

Quality Assessment

The quality assessment of the extracted studies was done through the utilization of the Newcastle-Ottawa Quality Assessment Scale (NOS) [10]. This scale is now considered a valid one to assess the risk of bias in non-randomized studies, including case-control and cohort studies. Similarly, this scale is also used for observational studies and was utilized for the quality assessment of our extracted studies. The quality assessment was also done by using the Jadad scale for RCTs [11].

Data Analysis

The DerSimonian and Laird random effects model was used to pool risk ratios (RR) with 95% confidence intervals (Cis) for categorical outcomes. The chi-square test and I2 statistic were used to assess heterogeneity among studies. P < 0.10 was considered statistically significant for the chi-square test. The analysis was conducted in Review Manager (RevMan, Version 5.4; The Cochrane Collaboration, Copenhagen, Denmark).

Results

Search Results

**Figure 1:**
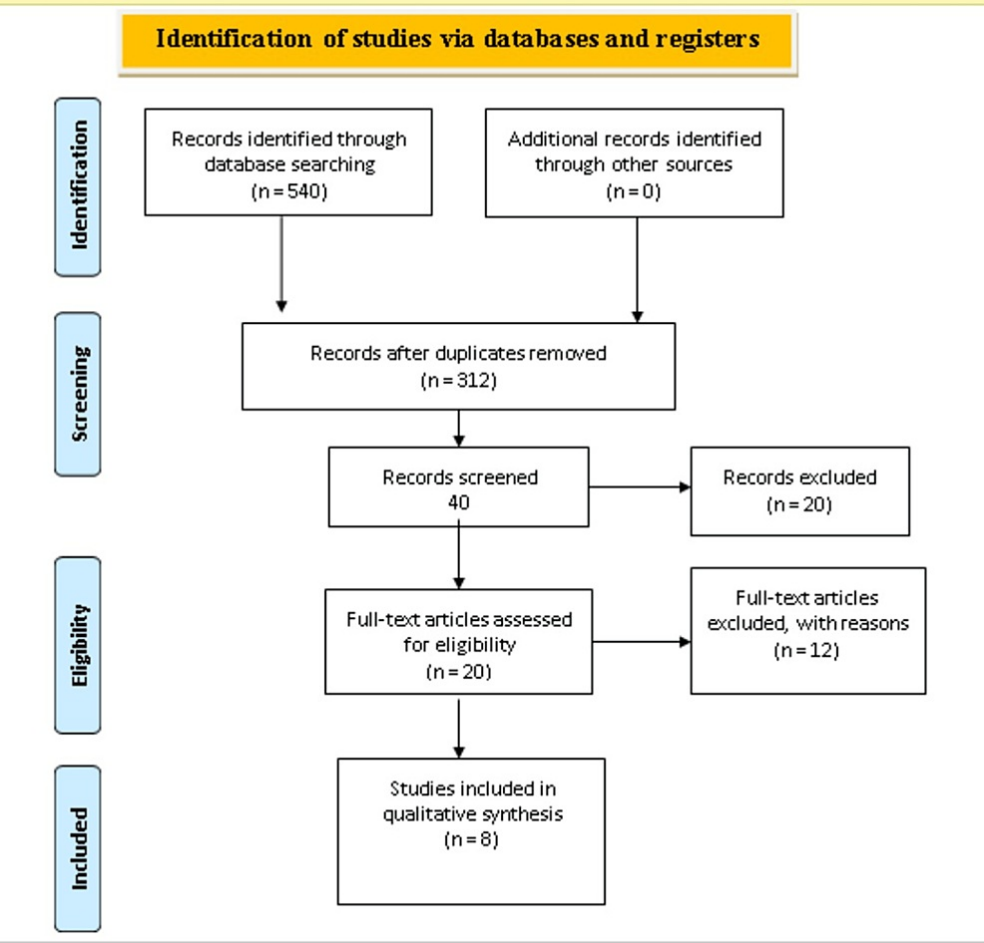
PRISMA flowchart of study selection. PRISMA: Preferred reporting of systematic reviews and meta-analyses Studies with duplication, lack of variables of interest and of low quality on quality assessment were excluded.

With an initial search, about 782 articles of interest were retrieved. After the removal of duplication and irrelevant studies (154), finally, 30 studies were assessed for eligibility, and only eight studies fell short of our criteria of research question and quality. Most of the studies were retrospective cohort studies, while randomized controlled trials (RCT) were also included. The PRISMA flow chart for the selection of the final eight studies [12-19] is given in Figure 1.

Population Characteristics and Outcomes

**Figure 2:**
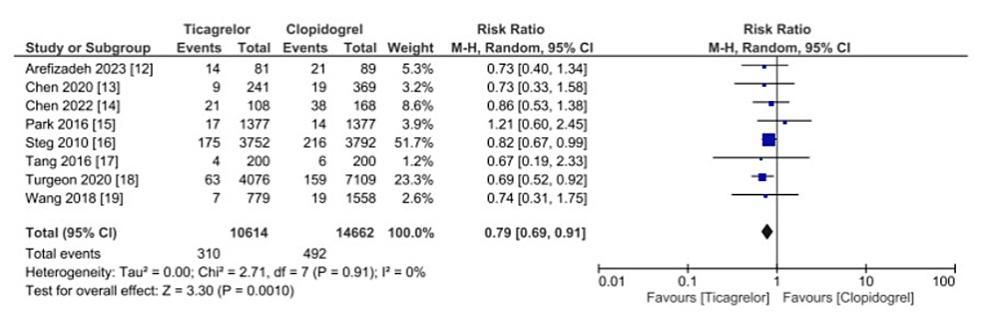
Comparative effectiveness of Ticagrelor vs. Clopidogrel in reporting all-cause mortality among patients who underwent percutaneous coronary intervention for acute coronary syndrome References: [12-19] CI: confidence interval; SE: standard error

All eight studies (25,276 patients) reported data on all-cause mortality, as shown in Figure 2. Ticagrelor was significantly superior to Clopidogrel (RR 0.79, 95% CI 0.69-0.91, p = 0.001). No heterogeneity was detected (I2 = 0%).

**Figure 3:**
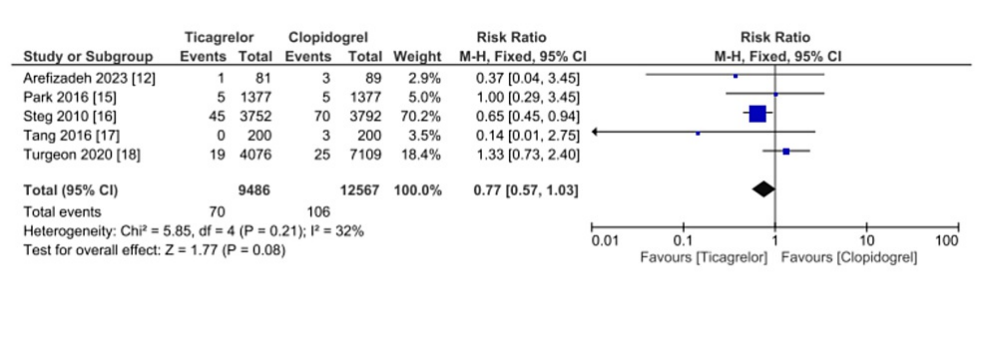
Comparative effectiveness of Ticagrelor vs. Clopidogrel in reporting stent thrombosis among patients who underwent percutaneous coronary intervention for acute coronary syndrome References: [12,15-18] CI: confidence interval; SE: standard error

Five studies (22,053 patients) reported an incidence of stent thrombosis, as shown in Figure 3. The two regimens were comparable in terms of risk of stent thrombosis (RR 0.77, 95% CI 0.57-1.03, p = 0.08). Significant heterogeneity was not found (I2 = 32%).

**Figure 4:**
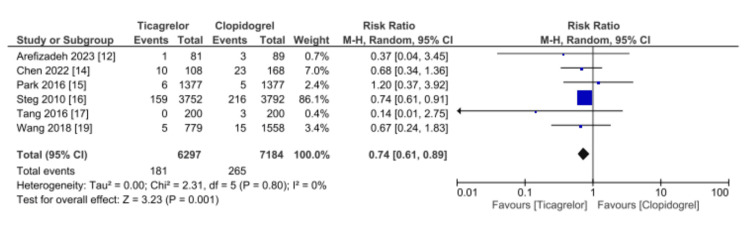
Comparative effectiveness of ticagrelor vs. clopidogrel in reporting myocardial infarction among patients who underwent percutaneous coronary intervention for acute coronary syndrome References: [12,14,15-17,19] CI: confidence interval; SE: standard error

Data on the occurrence of MI following percutaneous coronary intervention was reported by six studies (13,481 patients), as shown in Figure 4. The risk of MI was significantly lower with ticagrelor compared to clopidogrel (RR 0.74, 95% CI 0.61-0.89, p = 0.001). No heterogeneity was detected (I2 = 0%).

**Figure 5:**
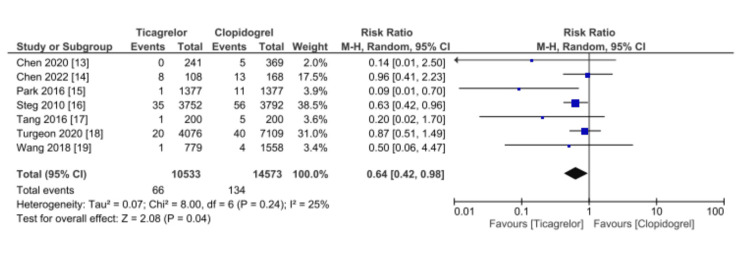
Comparative effectiveness of Ticagrelor vs. Clopidogrel in reporting stroke cases among patients who underwent percutaneous coronary intervention for acute coronary syndrome References: [13-19] CI: confidence interval; SE: standard error

Seven studies (25,106 patients) reported an incidence of stroke, as shown in Figure 5. Ticagrelor was significantly superior to Clopidogrel (RR 0.64, 95% CI 0.42-0.98, p = 0.04). Significant heterogeneity was not found (I2 = 25%).

**Figure 6:**
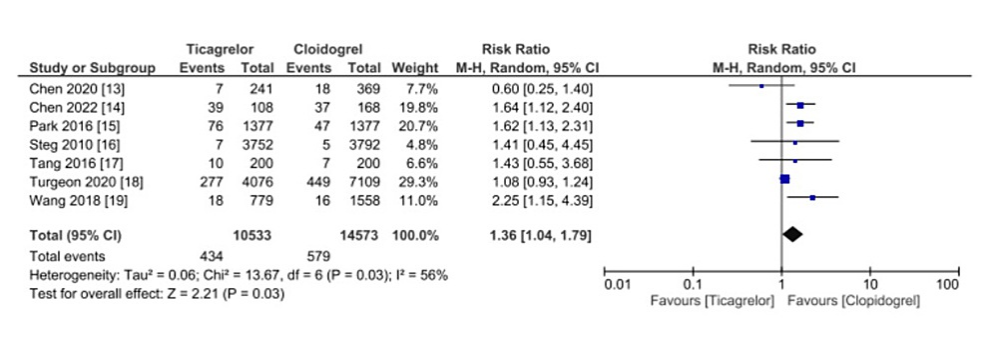
Comparative safety of Ticagrelor vs. Clopidogrel in reporting bleeding events among patients underwent percutaneous coronary intervention for acute coronary syndrome References: [13-19] CI: confidence interval; SE: standard error

Data on bleeding events was reported by seven studies, as shown in Figure 6. Clopidogrel was significantly superior to Ticagrelor (RR 1.36, 95% CI 1.04-1.79, p = 0.03). Significant heterogeneity was found (I2 = 56%).

Discussion

To prevent vascular complications in the post-ACS scenario, antiplatelet management is one of the key components of therapy. Clopidogrel was the previous drug of choice, but the requirement of hepatic metabolism resulted in a delayed onset as well as a longer duration of action. Furthermore, its hepatic metabolism might even contribute to resistance [20]. This has led to the development of ticagrelor, which works reversibly through adenosine-mediated vasoactive activity. This meta-analysis, based on eight studies, suggested that Ticagrelor was superior to Clopidogrel in terms of reducing all-cause mortality, M.I., and occurrence of stroke; however, the stent thrombosis events were comparable between these two groups. In the same way, the bleeding events were found to be higher among the Ticagrelor group as compared to the Clopidogrel group. 

Our study compared the merits of Ticagrelor and Clopidogrel using four different merits: all-cause mortality, incidences of stent thrombosis, M.I., and stroke. Our study showed Ticagrelor to be significantly superior in terms of all-cause mortality. A study by Cannon et al. showed similar results [21], and it corroborates our hypothesis as well. Furthermore, the study by James et al. showed that, even in patients who were managed conservatively, Ticagrelor reduced the risk of all-cause mortality better when compared with Clopidogrel [22]. All these studies were in favor of using Ticagrelor in terms of reducing all-cause mortality, and these findings are in concordance with what we have studied in our meta-analysis.

The risk of stent thrombosis was comparable for patients using both drugs in our study. This coincides with the study by James et al. but is contrary to the study by Cannon et al., which showed a significantly lower risk in patients using Ticagrelor [21,22]. The risk of M.I. was also shown to have significantly decreased. Similar results were reported by Cannon et al. [21]. However, James et al. showed no significant difference between the two groups. This could be due to the fact that these patients did not undergo any invasive procedures [22]. All these studies were in favor of using Ticagrelor in terms of reducing the risk of M.I., and these findings are in concordance with what we have studied in our meta-analysis.

Similarly, the risk of stroke was also reduced in patients taking Ticagrelor. This is in contrast to the studies by Cannon et al. and James et al., which showed no significant difference between the two drugs [21,22]. Our final parameter under study was bleeding events. This was the only parameter in which Clopidogrel was superior to Ticagrelor in our study. The study by Berwanger, however, showed that switching to Ticagrelor was not inferior to Clopidogrel in terms of the risk of bleeding [23]. Similarly, the studies by Cannon et al. as well as James et al. did not show any major difference between the two groups [21,22].

High on-treatment platelet reactivity (HTPR) is accompanied by an increased risk of adverse outcomes. A study by Li et al. showed that in patients with AMI or coronary artery ISR exhibiting HTPR after standard Clopidogrel treatment, Ticagrelor is significantly more effective compared with high-dose Clopidogrel in overcoming HTPR [24]. A study by Alexopoulus et al. showed similar results when done in patients undergoing fibrinolysis [25]. However, a study by Winter et al. concluded that, in patients presenting with STEMI, loading with Ticagrelor did not improve the angiographic and electrocardiographic parameters of myocardial reperfusion significantly, as compared to Clopidogrel [26].

This study has several limitations, like the small sample size of RCTs included in our meta-analysis. Similarly, the sample size taken from each of the included RCTs was also small. In the same way, the follow-up period of the patients after percutaneous coronary intervention was also variable among the different studies included in our meta-analysis. Similarly, patients with possible adverse drug events and discontinuation of the therapy were not included. All these above-mentioned points of limitations open the gates for more research.

## Conclusions

In short, the results of our study support the use of ticagrelor in patients undergoing percutaneous coronary intervention for acute coronary syndrome. A lower rate of all-cause mortality and a significantly lower risk of post-PCI myocardial infarction as well as stroke are all votes of confidence in this decision. The one parameter of bleeding events still needs more research, as it is a wide parameter and needs specific, detailed study.
